# Web-Based Patient-Reported Outcomes Using the International Consortium for Health Outcome Measurement Dataset in a Major German University Hospital: Observational Study

**DOI:** 10.2196/11373

**Published:** 2018-12-20

**Authors:** Maria M Karsten, Dorothee Speiser, Claudia Hartmann, Nele Zeuschner, Kai Lippold, Verena Kiver, Peter Gocke, Valerie Kirchberger, Jens-Uwe Blohmer

**Affiliations:** 1 Klinik für Gynäkologie mit Brustzentrum Charité Universitätsmedizin Berlin Berlin Germany; 2 Ärztliches Direktorat Charité Universitätsmedizin Berlin Berlin Germany; 3 Geschäftsstelle Vorstand Charité Universitätsmedizin Berlin Berlin Germany; 4 Stabstelle Digitale Transformation Charité Universitätsmedizin Berlin Berlin Germany; 5 Value-Based Healthcare Geschäftsbereich Strategische Unternehmensentwicklung Charité Universitätsmedizin Berlin Berlin Germany

**Keywords:** breast cancer, International Council Health Outcome Measurement, mobile phone, patient-reported outcomes

## Abstract

**Background:**

Collecting patient-reported outcome (PRO) data systematically enables objective evaluation of treatment and its related outcomes. Using disease-specific questionnaires developed by the International Consortium for Health Outcome Measurement (ICHOM) allows for comparison between physicians, hospitals, and even different countries.

**Objective:**

This pilot project aimed to establish a digital system to measure PROs for new patients with breast cancer who attended the Charité Breast Center This approach should serve as a blueprint to further expand the PRO measurement to other disease entities and departments.

**Methods:**

In November 2016, we implemented a Web-based system to collect PRO data at Charité Breast Center using the ICHOM dataset. All new patients at the Breast Center were enrolled and answered a predefined set of questions using a tablet computer. Once they started their treatment at Charité, automated emails were sent to the patients at predefined treatment points. Those emails contained a Web-based link through which they could access and answer questionnaires.

**Results:**

By now, 541 patients have been enrolled and 2470 questionnaires initiated. Overall, 9.4% (51/541) of the patients were under the age of 40 years, 49.7% (269/541) between 40 and 60 years, 39.6% (214/541) between 60 and 80 years, and 1.3% (7/541) over the age of 80 years. The average return rate of questionnaires was 67.0%. When asked about the preference regarding paper versus Web-based questionnaires, 6.0% (8/134) of the patients between 50 and 60 years, 6.0% (9/150) between 60 and 70 years, and 12.7% (9/71) over the age of 70 years preferred paper versions.

**Conclusions:**

Measuring PRO in patients with breast cancer in an automated electronic version is possible across all age ranges while simultaneously achieving a high return rate.

## Introduction

In Germany, every year, 70,000 women receive the diagnosis of breast cancer. Almost 30% are under the age of 55 years. Due to improved screening and treatment modalities, there has been a significant improvement in overall survival in the last decade [1-3]. Breast cancer-specific mortality in Europe reduced from 17.90 women in 2002 to 15.20 per 100,000 women in 2012 [4]. However, survival gains are often associated with the loss of physical functioning; increased morbidity; and new challenges regarding the emotional, social, and financial aspects of life [5-8]. Therefore, this increase in life expectancy of patients with cancer must lead to increased scrutiny regarding the long-term side effects of new and existing cancer treatments [9,10]. An important aspect in evaluating effects of any therapies is patients’ voice and perception. This applies even more to patients with cancer. The best way to address this aspect is to use patient-reported outcome (PRO) measures. The US Food and Drug Agency describes PRO as “any report coming directly from patients about a health condition and its treatment” [11]. Nowadays, using electronic media, like smartphones and tablet computers, measuring PRO data is much easier and less time- and cost-consuming than it was in the past. The use of PRO data allows for the real-time evaluation of therapy concepts and monitoring of new treatments. At the same time, it is an easy way for a long-term follow-up. Increasingly complex therapies in medicine simultaneously require an increase in documentation—time missing in direct patient communication [12,13]. However, this time is essentially needed to adequately assess a patient’s situation and symptoms. PRO has been shown in multiple studies to help clinicians to adequately assess patient symptoms, save time for patient communication, and therefore improve patient care and even survival [14,15]. The aim of this study was to establish a PRO system in a major German university hospital.

## Methods

After obtaining ethics approval from the Charité Ethics Commission (EA 4/127/16), we implemented a Web-based system to collect PRO data at the Charité Breast Center. Data capturing started in November 2016. The PRO data collection was based on an international standard set for breast cancer outcome measures, which was developed by the International Consortium for Health Outcome Measurement (ICHOM). All new patients, who attended the breast clinic, were included in the PRO measurement. Afterward, those who had a diagnosis of breast cancer and received their treatment at Charité Breast Center were stratified into follow-up. After patients registered at the clinic, they were asked by the receptionist if they would be willing to participate and received a personal log-in after they signed consent. The waiting time until their appointment was used to answer the ICHOM questions as well as questions regarding their medical history on a tablet computer. The decision to use a tablet computer in the clinic setting was one of the comforts because it allowed the patients to continue sitting where they felt the most comfortable in the waiting area. The follow-up emails patients received were designed in a way that they could also be answered on a mobile device.

After the successful completion of the questionnaire, answers and calculated PRO scores were immediately available to treating physicians for the upcoming consultation. During that, treating physicians had the option to add missing clinical data. If they decide not to record the clinical data necessary, it was later added by support staff. Once patients entered specific care pathways, like chemotherapy or surgery, an automated process was started through which they received follow-up emails containing the access code to their individual PRO measurement questionnaires. The follow-up emails were sent 6 weeks after their first treatment, then every 3 months thereafter for 2 years. After the first 2 years are finished, they will receive a follow-up questionnaire every 6 months for another 3 years and after this only on a yearly basis.

From a technical standpoint, the system for PRO collection was installed on campus as an on-premise installation. It was therefore only available within the Charité network. The core system was supported by an additional patient portal, which acted as an outward facing tool, to interact with patients. The patient portal was hosted in a different environment to allow for access to the Web. It enables patients to complete questionnaires from home using a secure connection.

## Results

### Monthly Increase in the Number of Patients Who Participate in the Patient-Reported Outcome Measurement Since the Program Implementation

**Figure 1:**
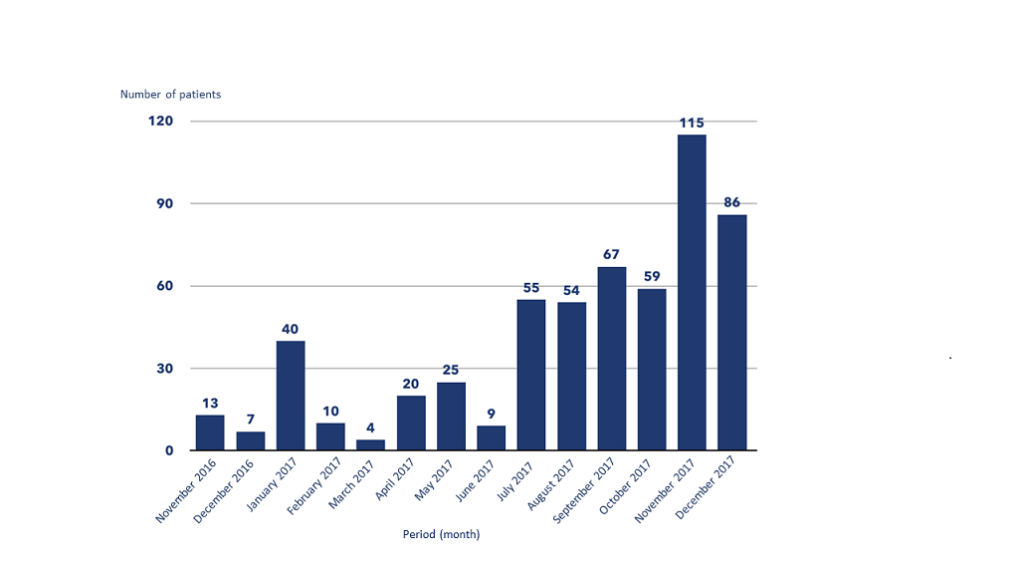
Monthly increase in patient numbers for patient-reported outcome measurement since implementation.

Figure 1 shows monthly increase in the number of patients who were entered into the PRO evaluation at the Breast Center and agreed to participate after its implementation in November 2016. After an initial increase in January 2017 with 40 patients, there was a decline in participation, with the lowest rate in March 2017, with only 4 patients included. From July 2017, there was a marked increase in patient numbers—a trend that continued from there on.

### Increase in Patients Numbers for Patient-Reported Outcome Measurement Compared With the Total Number of New Patients

Table 1 compares the number of new patients who entered into the PRO system with the total number of new patients seen at the Charité Breast Center. With the exemption of January 2017, where 34.4% (40/116) of the new patients were added to the PRO system, the percentage of all new patients stayed below 20% until July 2017, with the lowest percentage (4/166, 2.4%) in March 2017. After July 2017, there was a marked increase in adding new patients, almost continuously increasing and reaching its highest percentage in December 2017, with 73.5% (86/117) of all new patients included in the PRO system.

Table 2 shows patients’ characteristics regarding educational levels.

**Table 1:** Increase in patient numbers for patient-reported outcome measurement over time.

Period	New patients seen, n	Patients who participated in patient-reported outcome measurement, n (%)
November 2016	105	13 (12.3)
December 2016	90	7 (7.8)
January 2017	116	40 (34.4)
February 2017	124	10 (8.1)
March 2017	166	4 (2.4)
April 2017	104	20 (19.2)
May 2017	139	25 (17.9)
June 2017	112	9 (7.6)
July 2017	116	55 (47.4)
August 2017	108	54 (50.0)
September 2017	163	67 (41.1)
October 2017	93	59 (63.4)
November 2017	169	115 (68.0)
December 2017	117	86 (73.5)

**Table 2:** Level of academic training in all patients included in patient-reported outcome measurement (N=541).

Education level	Value, n (%)
Minimum German schooling requirement	201 (37.1)
Mid-level education	153 (28.2)
Higher level education	187 (34.5)

### Decline in Patient Numbers to Participate in the Long-Term Follow-Up

**Figure 2:**
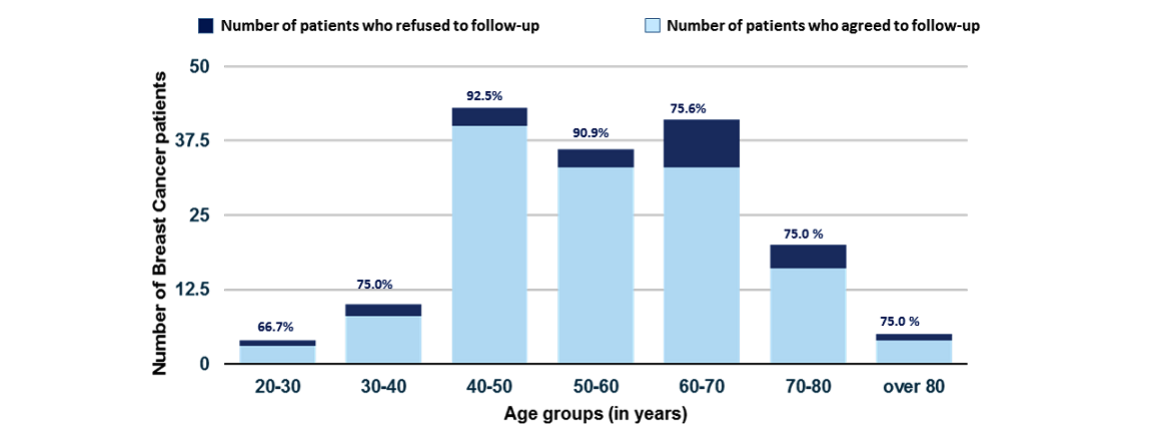
Patients who agreed to follow up versus who declined to follow up patient reported outcome measurement.

Figure 2 shows the percentage of breast cancer patients who agreed to participate in the electronic follow-up PRO measurement compared with those who did not want to participate. In the age group of 20-30 years, 66.67% (2/3) agreed to follow up; in the age group of 30-40 years, 75% (6/8) agreed to follow up. The highest number was seen in the age group of 40-50 years, with 90.91% (37/40) followed up; 75.76% (25/33) agreed to follow up in the age group 60-70 years. Participation levels were the same, with 75% in the age groups of 70-80 (12/16) and >80 (3/4) years.

### Return Rates of Questionnaires From Patients Participating in the Follow-Up Patient-Reported Outcome Measurement

**Figure 3:**
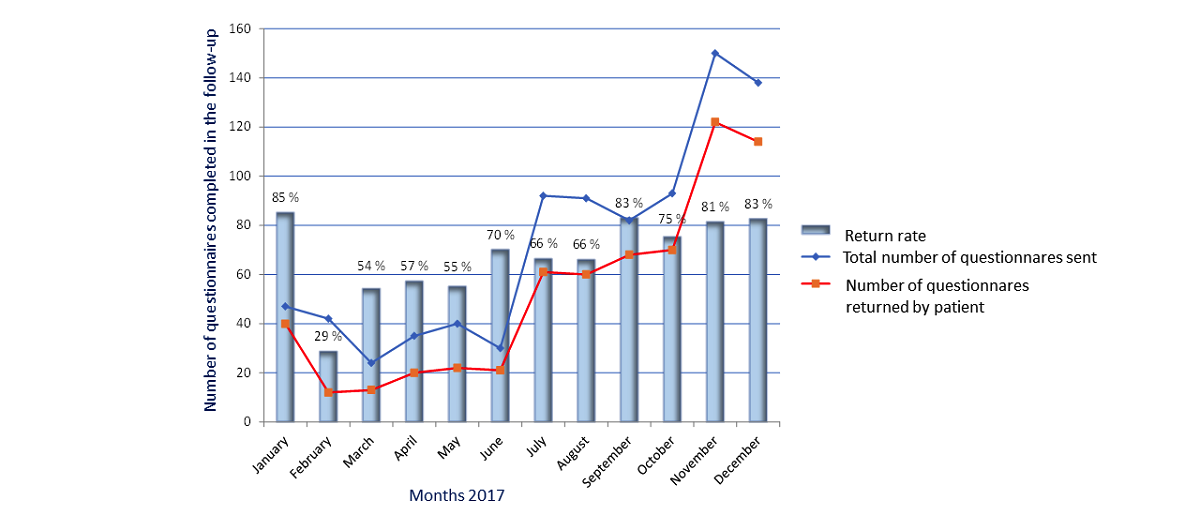
Return rates of the digital questionnaires completed by patients who participated in the long-term patient-reported outcome measurement.

Figure 3 shows the return rates of the online questionnaires completed by those patients who participated in the follow-up PRO measurement. After an initial drop to 28.6% (12/42) in February 2017, there was a continuous increase in the return rate from 54.2% (13/24) in March of 2017 to 82.9% (68/82) in September 2017, with then another slow decline in October 2017 to 75.3 (70/93). November and December 2017 showed steady return rates above 81.3% (122/150) and 82.6% (114/138), respectively.

### Preference for Using a Digital Way to Measure Patient-Reported Outcome Compared With a Paper-Based Version Across Age Groups

**Figure 4:**
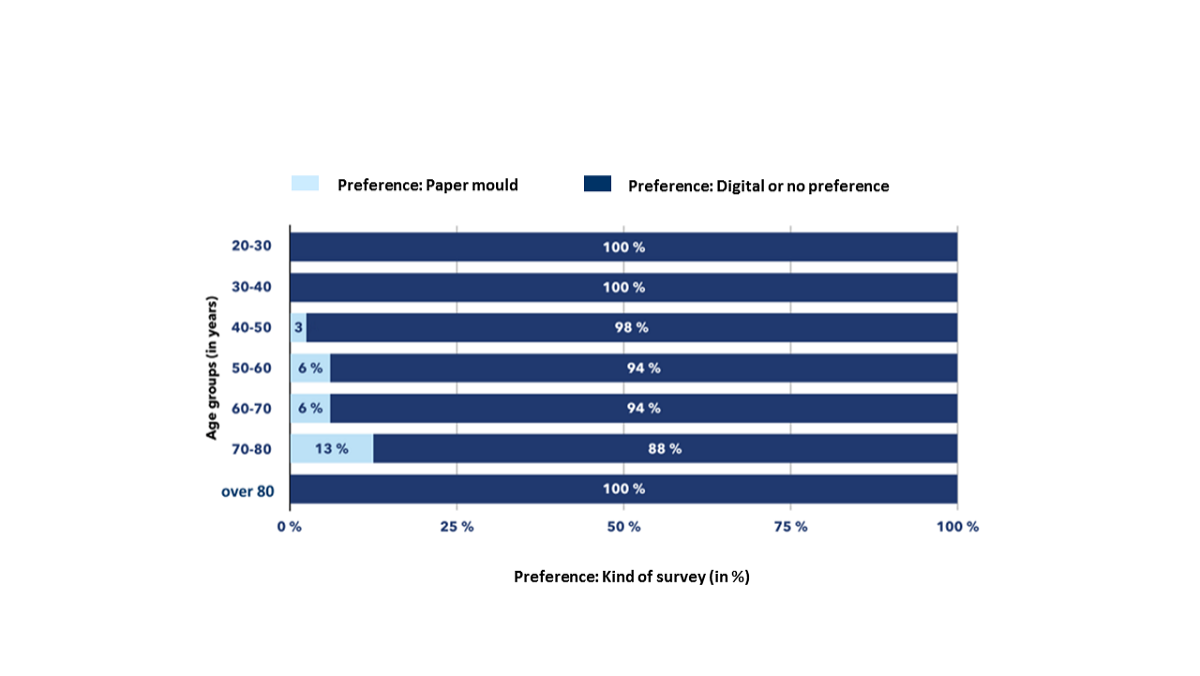
Preference for paper versus digital questionnaire according to age groups.

Figure 4 shows the percentage of patients who preferred a paper-based version of the questionnaire instead of a digital one. Notably, 100% (51/51) of the patients in the age groups of 20-40 years preferred a digital version, while 2.9% (7/244) of the patients in the age group of 40-50 years preferred a paper-based version. This increases to 5.9% (13/122) in the age group of 50-70 years and then further increases to 13.0% (3/23) in those aged 70-80 years. Above 80 years of age, there was a 100% (2/2) preference for paper-based questionnaires compared with digital questionnaires.

## Discussion

### Principal Findings

This study documents the successful implementation of measuring PROs using the ICHOM dataset for breast cancer in a German university hospital for the first time. During the implementation period, we made numerous observations. First, it takes at least 6 months to implement and establish a working system, get all key stakeholders to adopt it, while simultaneously solve those technical problems that arise during the implementation phase. Second, most patients, across all age groups, are willing to participate in the initial measurement as well as in the long-term follow-up. Third, contrary assumption, most patients, even those aged >60 years, prefer a digital survey over a paper-based way to answer the PRO questionnaires.

Our observation regarding the required timeframe to establish a successful electronic PRO system is matched by previous publications [16-18]. A crucial point in establishing a successful digital program is to explain and educate all health care providers who will be involved in the collection of PROs about the purpose and benefit of PRO. While there is increasing interest and knowledge about PRO measurement, there are concerns regarding workflow, increase in workload due to the additional measurements, as well as data overload and creation of additional needs [19-21]. These findings are mirrored in our low patient accrual data in the first 6 months after the implementation. It took this time to train, educate, and convince all involved staff members from front desk staff to treating physicians. Once this barrier was broken, there was a steady increase in patients with breast cancer who agreed to participate in the follow-up PRO measurement. Only a small percentage of patients declined to participate in the PRO follow-up. The highest percentage of patients who agreed to follow up was in the age group of 40-50 years, at an astonishing 90.91% (37/40). The lowest rate was found in the age group of 20-30 years, with only 66.67% (2/3) agreeing to follow up. This is in part because of the low numbers of patients we have in this age group. Previous work has shown that especially technology literacy plays a role in the extent to which patients are participating in electronic-based PRO measurement [22]. We did not gather information regarding technology literacy but asked a question regarding the level of education patients had because there have been data showing a correlation between the level of education and the willingness to participate in Web-based data collection as well as the effects regarding PRO-based interventions [15,23]. As shown in Table 2, there was a fairly even distribution of educational levels in our patient population. Also, the access and ability to use a computer, smartphone, or smartwatch is a prerequisite, and therefore, patients who had neither of these and thereby often did not have an email address were not able to participate in the study. This could be a potential bias, especially regarding the return rates.

In addition to the finding that it is possible to establish a successful PRO measurement program at a German breast center integrated into the routine clinical workflow, this work showed for the first time that the majority of patients with breast cancer treated in a German university hospital preferred a digital survey. Patients did not want to fill out questionnaires in a paper and pencil-based version—despite contrary believes. In addition to this interesting finding, we were able to show that we can simultaneously achieve a high adherence rate even in the long-term follow-up. Similar observations have been made previously in the United States, for example, at Memorial Sloan Kettering Cancer Center [24], the University of North Carolina [25], or Group Health Cooperative in Seattle [26].

### Limitations

Since this is a retrospective analysis of an implementation trial, it is not without limitations. In the beginning, reasons for decline in the number of patients participating was not systematically collected. Since this systematic approach was started only at a later point, we currently do not have enough data on this matter but are collecting them now. Also, we did not have enough resources to be able to contact those patients who decided not to continue in the follow-up. This point and the first point are important aspects, and we are currently addressing both and plan to publish the results in a following publication.

### Comparison With Prior Work

While there is an increasing interest in the potential of PRO measurements in almost all disease entities [27-30], there is still a lack of standards regarding what to measure and how to measure it. The ICHOM initiative has therefore created a working group for a wide range of diseases with the goal to establish standard sets to compare outcomes between different providers, hospitals, and even countries [31]. This is the first published work to show that the implementation of one of their standard sets—in our case for breast cancer—is feasible and lays the foundation for further improvements in the complex care of patients with breast cancer.

### Conclusions

The goal of this pilot trial was to create a template on how to establish a successful Web-based PRO measurement system at a German university hospital, setting the stage for what to expect and showing that it is possible to measure PRO in a digital manner in patients with breast cancer of all age groups.

## Abbreviations

ICHOM: International Consortium for Health Outcome Measurement
PRO: patient-reported outcomes
